# Overexpression of *SlOFP20* in Tomato Affects Plant Growth, Chlorophyll Accumulation, and Leaf Senescence

**DOI:** 10.3389/fpls.2019.01510

**Published:** 2019-11-29

**Authors:** Shengen Zhou, Xin Cheng, Fenfen Li, Panpan Feng, Gongling Hu, Guoping Chen, Qiaoli Xie, Zongli Hu

**Affiliations:** Laboratory of Molecular Biology of Tomato, Bioengineering College, Chongqing University, Chongqing, China

**Keywords:** OVATE family protein, SlOFP20, chlorophyll, sugar metabolism, leaf senescence

## Abstract

Previous studies have shown that OVATE family proteins (OFPs) participate in various aspects of plant growth and development. How OFPs affect leaf chlorophyll accumulation and leaf senescence has not been reported yet. Here, we found that overexpression of *SlOFP20* in tomato not only impacted plant architecture but also enhanced the leaf chlorophyll accumulation and retarded leaf senescence. Gene expression analysis of *SlGLK1, SlGLK2, and HY5*, encoding transcription factors that are putatively involved in chloroplast development and chlorophyll levels, were significantly up-regulated in *SlOFP20-OE* lines. Both chlorophyll biosynthesis and degradation genes were distinctly regulated in transgenic plants. Moreover, *SlOFP20-OE* plants accumulated more starch and soluble sugar than wild-type plants, indicating that an increased chlorophyll content conferred some higher photosynthetic performance in *SlOFP20-OE* plants. Furthermore, The levels of leaf senescence-related indexes, such as hydrogen peroxide, malondialdehyde, and antioxidant enzymes activities, were differently altered, too. *SlOFP20* overexpression repressed the expression of senescence-related genes, *SAG12*, *RAV1*, and *WRKY53*. Moreover, abscisic acid and ethylene synthesis genes were down-regulated in transgenic lines. These results provide new insights into how SlOFP20 regulates chlorophyll accumulation and leaf senescence.

## Introduction

Chlorophylls, in complex with their binding proteins, serve primary functions in photosynthesis through trapping light energy and transferring it to the reaction centers of photosystems (Meskauskiene et al., 2001; Goslings et al., 2004). Many factors affect the accumulation of chlorophyll, such as environmental conditions, the structural integrity of the chloroplast, plant hormones, as well as the expression levels of transcription factors and structural genes within the chlorophyll biosynthesis pathway (Meng et al., 2018). To date, some transcription factors have been reported to control chloroplast development and chlorophyll synthesis. For example, the transcription factor SlGLK2 determines plastid and chlorophyll levels *via* enhancing photosynthesis gene expression and chloroplast development (Fitter et al., 2002; Powell et al., 2012; Nguyen et al., 2014). TKN2 and TKN4, two Class I KNOTTED1-LIKE HOMEOBOX (KNOX) proteins, induce the expression of *SlGLK2* and *SlAPRR2-LIKE* genes to promote fruit chloroplast development in tomato (Nadakuduti et al., 2014). Triple mutant plants of *GRAS* gene family members, namely *SCL6-II, SCL6-III*, and *SCL6-IV*, exhibit increased chlorophyll accumulation in leaves (Wang et al., 2010). Moreover, phytohormones participate in chloroplast development and chlorophyll synthesis. For instance, cytokinins (CKs) aﬀect chloroplast function through regulating photosynthetic performance (Cortleven and Schmulling, 2015) and triggering chloroplast-related genes (Cortleven et al., 2016). The abscisic acid-deficient mutant, *high-pigment 3*, accumulates more carotenoids and chlorophylls in leaves and fruits (Galpaz et al., 2008). Previous research has suggested that detached roots activate chlorophyll biosynthesis through a reduction in auxin signaling, reflecting the repressive effect of auxin on root greening (Kobayashi et al., 2012).

Leaf senescence occurs at the final stage of leaf development and involves a series of changes at the molecular, cellular and phenotypic levels. Senescence is initiated by characteristic degenerative processes, such as chlorophyll degradation and macromolecule breakdown, and particularly the recycling of nutrients to actively growing tissues or storage organs (Gan and Amasino, 1997). Developmentally regulated metabolic pathways are typically inﬂuenced by transcription factors (Yin et al., 2016). For instance, suppression of *SlNAP2* expression in tomato delays leaf senescence but boosts fruit yield and sugar content (Ma et al., 2018). However, the *wrky54*/*wrky70* double mutant distinctly shows premature senescence and chlorophyll degradation (Besseau et al., 2012). In addition, phytohormones can either induce or inhibit leaf senescence. Ethylene-insensitive mutants, such as *etr1-1* (Grbić and Bleecker, 1995) and *ein2/ore3* (Oh et al., 1997), exhibit a delayed senescence phenotype. Exogenous application of abscisic acid (ABA) rapidly induces the senescence syndrome and expression of several senescence-associated genes (SAGs) (Gepstein and Thimann, 1980; Weaver et al., 1998). Increased CKs production could slow down leaf senescence, whereas reduced endogenous CK levels may result in premature senescence (Zhang et al., 2010; Qin et al., 2011).

Brassinosteroids (BRs) are steroid hormones of plants that were identiﬁed in the 1970s because of their strong growth-promoting capacities (Mitchell et al., 1970; Grove et al., 1979). BRs regulate cell elongation, cell division, and cell differentiation and function throughout plant development in various developmental programs, including seedling development in the light and dark, adult shoot and root growth, ﬂowering, fruit development, and senescence (Clouse, 2011). Extensive studies using genetic, molecular, and proteomic approaches have been applied to reveal how BRs modulate a wide range of plant growth and development processes. However, the effects of BRs on tomato growth and development are largely unknown, as well as the underlying molecular mechanism. Recently, ectopic expression of *BZR1-1D*, encoding a transcription factor in BR signaling, enhances carotenoid accumulation and fruit quality attributes in tomato (Liu et al., 2014). *DWARF* overexpression induces alteration in phytohormone homeostasis, development, architecture and carotenoid accumulation in tomato (Li et al., 2016). BR enhances chilling tolerance through a signaling cascade involving *RBOH1* (*RESPIRATORY BURST OXIDASE HOMOLOG1*), *GRX* (*GLUTAREDOXIN*) genes, and 2-Cys Prx (2-cysteine peroxiredoxin) in tomato (Xia et al., 2018).

The *OVATE* gene was originally identified as a primary quantitative trait locus that controls fruit shape in tomato (Liu et al., 2002). OVATE family proteins (OFPs) control multiple aspects of plant growth and development. It has been suggested that AtOFP1 functions as a transcriptional repressor and regulates cell elongation (Wang et al., 2007). AtOFP5 negatively regulates the activity of a BLH1-KNAT3 complex during early embryo sac development (Pagnussat et al., 2007). More recently, co-expression of *MuMADS1* and *MaOFP1* in the *ovate* mutant could compensate for fruit shape and inferior qualities (Liu et al., 2018). Overexpression of *OsOFP19* caused a semi-dwarf stature with thicker leaves and stronger culms and roots (Yang et al., 2018).

Here, we report the functional characterization of tomato *SlOFP20*, an *OFP* family member that is a homolog of the *Arabidopsis AtOFP1* and rice *OsOFP19*. Some quite recent research indicates that *SlOFP20* control fruit shape, and down-regulation of *SlOFP20* in WT tomato does not generate visible phenotypes (Wu et al., 2018). In our study, overexpression of *SlOFP20* produced pleiotropic phenotypes. We focused on the accumulation of chlorophyll and delay of leaf senescence caused by *SlOFP20* overexpression. Our data expand our understanding of the functions of OFPs in the regulation mechanisms of chlorophyll accumulation and leaf senescence.

## Materials and Methods

### Plant Materials and Growth Conditions

The WT tomato (*Solanum lycopersicon* Mill. cv. Ailsa Craig) and T2 *SlOFP20*-OE transgenic plants were used in our research and grown in a greenhouse with the following conditions (16-h-day/8-h-night cycle, 28°C/18°C day/night temperature). To examine the response of *SlOFP20* to phytohormones, 50 µM gibberellic acid (GA_3_), 50 µM indole-3-acetic acid, 100 µM ABA (0.0264 g ABA was dissolved in a small amount of methanol, followed by adding distilled water to 1 l), 50 µM 1-aminocyclopropane-1-carboxylate (ACC) or distilled water (control) were used to treat 35-day-old WT tomato seedlings. For each case, individual plants were used for each treatment with three biological replicates. After treatments for 0, 1, 2, 4, 8, 12, and 24 h, the third leaves from the top of the WT tomato seedlings were collected. For salinity stress treatment, the roots of tomato seedlings were immersed in a solution with 200 mM sodium chloride for 0, 1, 2, 4, 8, 12, and 24 h, then leaves and roots from the treated seedlings and control plants were collected. For dehydration experiments, the whole tomato seedlings were carefully pulled out of the pots, washed gently with water to remove soil and left on a piece of dry ﬁlter paper under dim light at 25 ± 1°C. For low temperature experiments, the whole potted tomato seedlings were incubated at 4°C for 0, 1, 4, 8, 12, and 24 h, after which the leaves were harvested. All these samples were instantly frozen with liquid nitrogen and kept at –80°C.

### Construction of *SlOFP20* Over-Expression Vector and Plant Transformation

To obtain *SlOFP20* overexpression transgenic lines, the full-length *SlOFP20* coding sequence was amplified using WT tomato complementary DNA with *SlOFP20*-F and *SlOFP20*-R primers, which tailed with *Xba* I and *Sac* I restriction sites at the 5′end, respectively. The ampliﬁed products were digested and cloned into the plant binary vector pBI121 placed under the control of CaMV 35S promoter. The resulting expression vector was transferred into *Agrobacterium* LBA4404 strain, and the positive LBA4404 strain was transferred into WT tomato cotyledon explants. Transformed lines were selected for kanamycin resistance and then analyzed by polymerase chain reaction (PCR) to determine the presence of T-DNA using the primers *NPTII*-F/R.

### Gene Expression Analysis

Gene expression analysis was performed according to our previous report (Zhou et al., 2018). Primers used for quantitative real-time (RT)-PCR are listed in **Supplementary Table S1**.

### Stoma Morphology and Anatomical Analyses of the Leaves

The lower epidermis from WT and OE3 mature leaves were torn off and observed under a Nikon E100 microscope. The middle parts of WT and OE3 mature leaves were cut and fixed by FAA [70% ethanol/acetic acid/formaldehyde (18:1:1)]. Then the dehydration, ﬁxation, sectioning, dewaxing and staining by safranin and fast green (Bryan, 1955) were performed to prepare the cross sections of WT and OE3 transgenic plant leaves, which were visualized under a Nikon E100 microscope and photographed.

### Quantitative Analysis of Chlorophyll Content

Chlorophyll content was determined in mature leaves of WT and *SlOFP20*-OE plants according to the methods described by Arnon (1949).

### Sugar Content Analysis

Leaves from WT and *SlOFP20*-OE plants were collected and immersed in 95% ethanol to destain for at least 24 h, then the thoroughly bleached leaves were stained with an I_2_/KI solution for 20 min. For measurement of starch and soluble sugar contents, 0.5 g fresh leaves of WT and *SlOFP20*-OE transgenic lines were pounded to powder with liquid nitrogen, extracted with 5 ml 80% ethanol for 30 min in a water-bath at 80°C, and then centrifuged 3,500 g for 10 min at room temperature. The supernatant was transferred to a 50 ml centrifuge tube, which was used to measure soluble sugar. These steps were repeated twice. Two milliliter distilled water was added to the residue, which then placed in a boiling water bath for 15 min. After cooling, added 2 ml 9.2 M perchloric acid, stirred for 15 min, mixed with 4 ml distilled water, and centrifuged 4,000 g for 10 min, the supernatant was transferred to a 50 ml centrifuge tube, 4.2 M perchloric acid was added to the residue again, stirred for 15 min, mixed with 5 ml distilled water, centrifuged 4,000 g for 10 min, the supernatant was merged into the centrifuge tube, which was used to measure starch content. The anthrone–sulfuric acid method was used to measure starch and soluble sugar contents (Leng et al., 2016). Three independent experiments were carried out and results expressed as mean ± SD of all replicates.

### Analysis of Hydrogen Peroxide Content, Malondialdehyde Content, and Antioxidant Enzyme Activities

Hydrogen peroxide (H_2_O_2_) content was determined according to the method of Sergiev et al. (1997). Malondialdehyde (MDA) content was estimated according to Zhu et al. (2015). The activity of superoxide dismutase (SOD) was determined according to the methods described by Beyer and Fridovich (1987). The activity of catalase (CAT) was determined according to the methods described by Rao et al. (1996). The activity of peroxidase (POD) was determined according to the methods described by Morohashi (2002).

### Statistical Analysis

All the experiments included three independent repeats. The signiﬁcant diﬀerence between WT and *SlOFP20*-OE lines was analyzed by Student's t-test (*P* < 0.05).

## Results

### The Response of *SlOFP20* to Various Hormone and Stress Treatments

**Figure 1:**
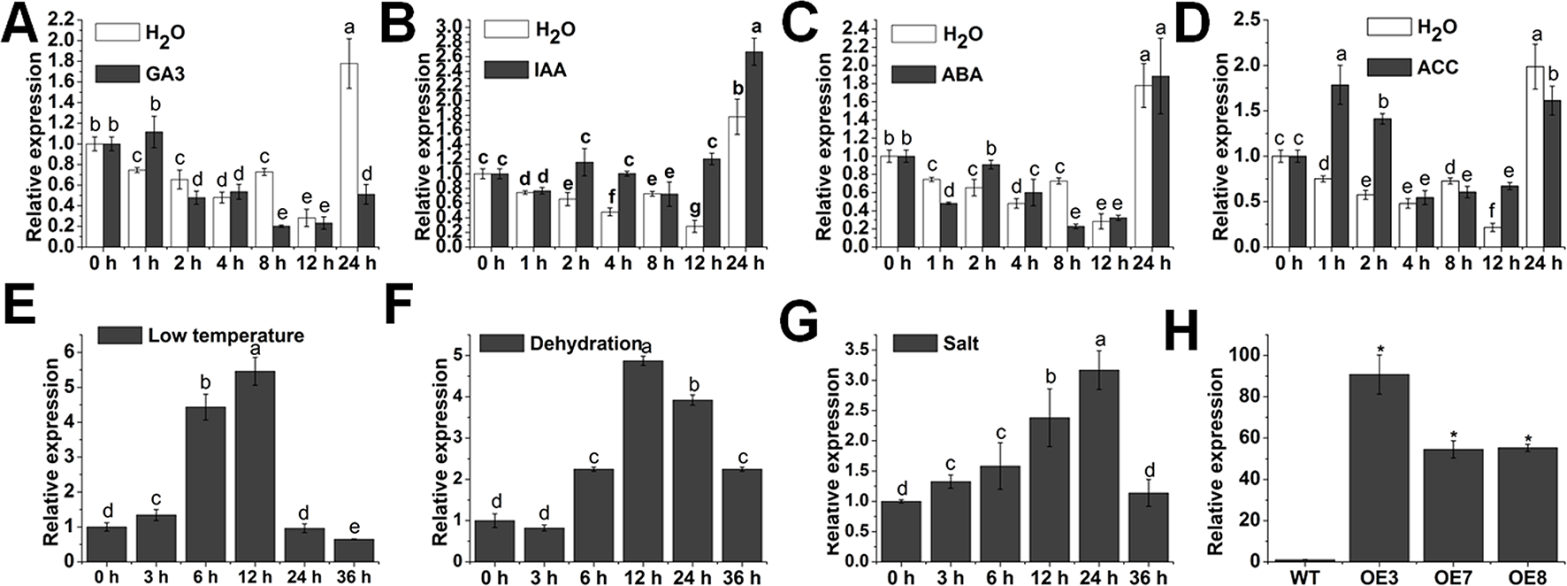
Expression patterns of *SlOFP20*. The expression level of *SlOFP20* in response to the phytohormones gibberellic acid **(A)**, indole-3-acetic acid **(B)**, abscisic acid **(C)**, and 1-aminocyclopropane-1-carboxylate **(D)**. The expression of *SlOFP20* under low temperature **(E)**, dehydration **(F)**, and salt stress **(G)**. Data are the means ± SD of three independent experiments. Signiﬁcant differences (*P* < 0.05) are denoted by different letters. Relative expression proﬁles of *SlOFP20* between WT and *SlOFP20*-OE lines. Data are the means ± SD of three independent experiments. Asterisks indicate a signiﬁcant difference (*P* < 0.05).

Phytohormones act as crucial regulators to coordinate multiple developmental processes and responses to environmental stresses (Bari and Jones, 2009). To obtain some clues of whether *SlOFP20* is involved in hormone signaling, we investigated the expression patterns of *SlOFP20* under different hormone treatments by quantitative RT-PCR technology (qRT-PCR). *SlOFP20* was significantly repressed by GA_3_ treatment at 8 and 24 h (**Figure 1A**). For indole-3-acetic acid, the transcript level of *SlOFP20* was induced from 2 to 4 h and 12 to 24 h, and no noticeable change at 8 h (**Figure 1B**). When treated with ABA, the expression of *SlOFP20* had no significant change until 8 h, and then declined sharply at 8 h, reached a similar level from 12 to 24 h. (**Figure 1C**) The *SlOFP20* messenger RNA (mRNA) was accumulated from 1 to 2 h and at 12 h after ACC treatment, and no apparent change at other time points (**Figure 1D**). These results indicated that *SlOFP20* may play an important role in plant hormone response and signal transduction. Rice OFP 6 has been suggested to confer resistance to drought and cold stresses (Ma et al., 2017). Except for this, few studies have been focused on the functional characterization of OFPs referring to stress response. To know whether *SlOFP20* participates in response to various abiotic stresses, qRT-PCR was used to detect the expression patterns of *SlOFP20* under different stress treatments. Under low-temperature conditions, the expression of *SlOFP20* was distinctly increased and peaked at 12 h and then declined (**Figure 1E**). *SlOFP20* mRNA was remarkably raised after dehydration treatments and peaked at 12 h, and then fell (**Figure 1F**). *SlOFP20* was signiﬁcantly induced after salt treatments and peaked at 24 h and then decreased (**Figure 1G**). These results give a further hint that *SlOFP20* may take part in response to abiotic stress in tomato.

To understand the biological function of *SlOFP20*, the plasmid 35S:*SlOFP20* was introduced into WT tomato AC^++^, the expression levels of *SlOFP20* in T2 transgenic lines were detected by qRT-PCR. The expression of *SlOFP20* was 90-, 54-, and 55-fold higher in the overexpression T2 lines OE3, OE7, and OE8, respectively, compared with WT plants (**Figure 1H**).

### Ectopic Expression of *SlOFP20* May Lead to Typical Brassinosteroids-Insensitive and Gibberellic Acid-Deﬁcient Phenotypes

A gain-of-function mutant of *AtOFP1* exhibited decreased lengths in all aerial organs, which was partially attributed to the deﬁciency in gibberellin biosynthesis (Wang et al., 2007). Overexpression of *OsOFP19* in rice modified plant architecture, including semi-dwarf stature with thicker leaves, stronger culms and roots and grain shape, by integrating the cell division pattern and BR signaling (Yang et al., 2018). In our study, we also observed that overexpression of *SlOFP20* in WT tomato resulted in a series of phenotypes related to plant vegetative growth.

**Figure 2:**
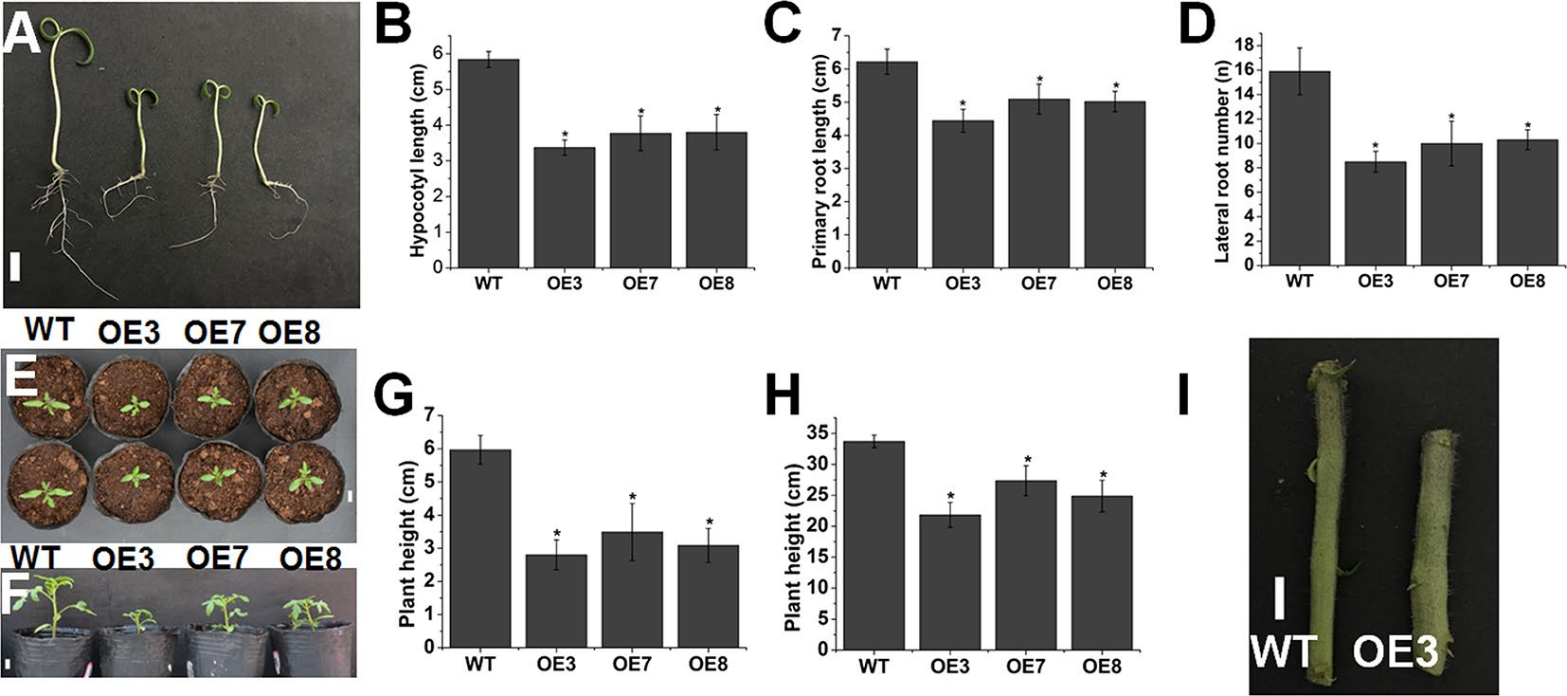
Overexpression of *SlOFP20* affected plant growth. **(A)** Seven-day-old seedlings of WT and *SlOFP20*-OE lines. Bar = 1 cm. **(B)** Hypocotyl lengths of 7-day-old WT and *SlOFP20*-OE seedlings. **(C)** The length of primary root in WT and *SlOFP20*-OE 7-day-old seedlings. **(D)** The number of lateral roots in WT and *SlOFP20*-OE 7-day-old seedlings. Error bars show the standard error between three biological replicates with 10 plants for each replicate performed. Asterisks indicate a signiﬁcant difference (*P* < 0.05). **(E)** Top view of cotyledons from 7-day-old WT and *SlOFP20*-OE lines. **(F)** Image of 30-day-old WT and *SlOFP20*-OE plants. Bar = 1 cm. **(G**, **H)**.Plant height of WT and *SlOFP20*-OE plants at 30 days **(G)** and 60 days **(H)** after sowing. Error bars show the standard error between three biological replicates with eight plants for each replicate performed. **(I)** Stems of WT and OE3 plants. Bar = 1 cm.

Compared with WT tomato seedlings, overexpression of *SlOFP20* led to decreased length of the primary root, hypocotyl and cotyledon and reduced number of lateral roots (**Figures 2A–E**). We also found kidney-shaped cotyledons in *SlOFP20*-OE transgenic plants (data not shown). In addition, overexpression of *SlOFP20* caused a dwarf phenotype (**Figure 2F**), we measured the plant height of WT and *SlOFP20-*OE transgenic lines at 30 and 60 days after sowing in pots under the same conditions, and the height of *SlOFP20*-OE transgenic plants was much shorter than WT tomato plants (**Figures 2G, H**). We also observed that the stem of *SlOFP20*-OE transgenic plants was much thicker when compared with WT tomato plants (**Figure 2I**).

**Figure 3:**
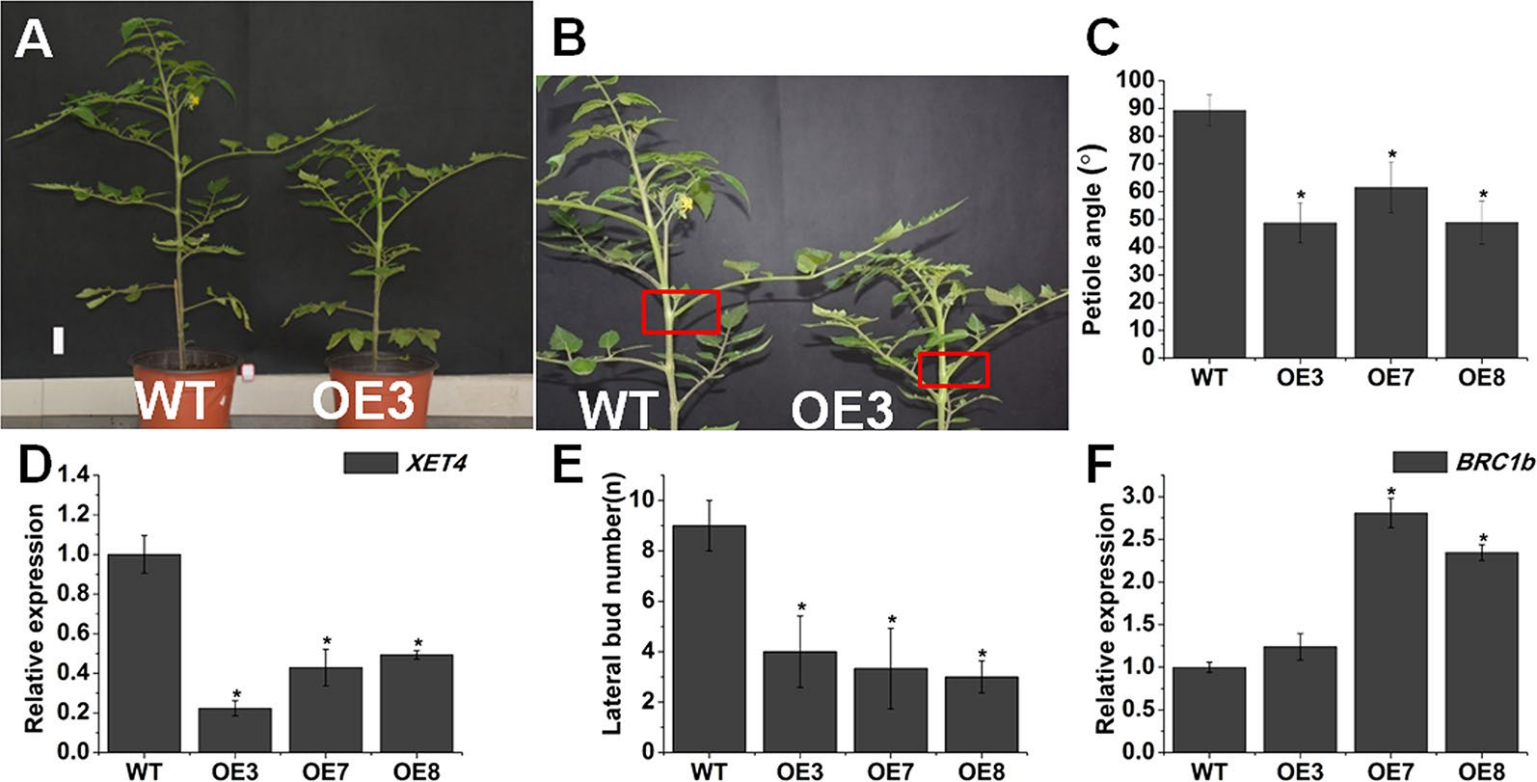
Overexpression of *SlOFP20* restricts leaf inclination and lateral bud outgrowth. **(A)** Picture of 60-day-old WT and OE3 plants. Bar = 1 cm. **(B)** The red boxes indicated the petiole angle of the 9th leaf. **(C)** The petiole angle was determined at 60 days in the ninth leaf with eight plants after sowing. Error bars show the standard error between three biological replicates. Asterisks indicate a signiﬁcant difference (*P* < 0.05). **(D)** The expression level of *XET4* in WT and *SlOFP20*-OE lines. **(E)** Lateral bud numbers of WT and transgenic plants. At least eight plants were measured for WT and each transgenic line. Error bars show the standard error between three biological replicates. Asterisks indicate a signiﬁcant difference (*P* < 0.05). **(F)** Expression analysis of *BRClb* in WT and transgenic plants. Each value represents the mean ± SE of three biological replicates. Asterisks indicate a signiﬁcant difference (*P* < 0.05).

The changes of endogenous BR level and BR signaling affect leaf angle and lateral bud initiation (Li et al., 2016). *SlOFP20*-OE plants also displayed upright leaves. The 9th leaf petiole angle of *SlOFP20*-OE plants was clearly reduced, in comparison with WT tomato plants (**Figures 3A–C**). We inferred that *SlOFP20* may affect leaf inclination through transcriptional regulation of the relevant genes. *OsXTR1*, which encodes a xyloglucan endotransglycosylase, is a cell wall-loosening enzyme necessary for cell elongation (Duan et al., 2006). Compared with WT tomato plants, the expression of *XET4*, the homologous gene of *OsXTR1* in tomato, was significantly down-regulated in *SlOFP20*-OE plants. Furthermore, *SlOFP20*-OE transgenic plants had few branches throughout the life cycle. Compared with WT tomato plants, the number of branches reduced by at least 50% in overexpression lines (**Figure 3E**). *SlBRC1b* acts as a suppressor of shoot branching in tomato (Martín-Trillo et al., 2011). Gene expression analysis suggested that the expression of *BRC1b* was increased in *SlOFP20* overexpression lines when compared with WT plants (**Figure 3F**).

**Figure 4:**
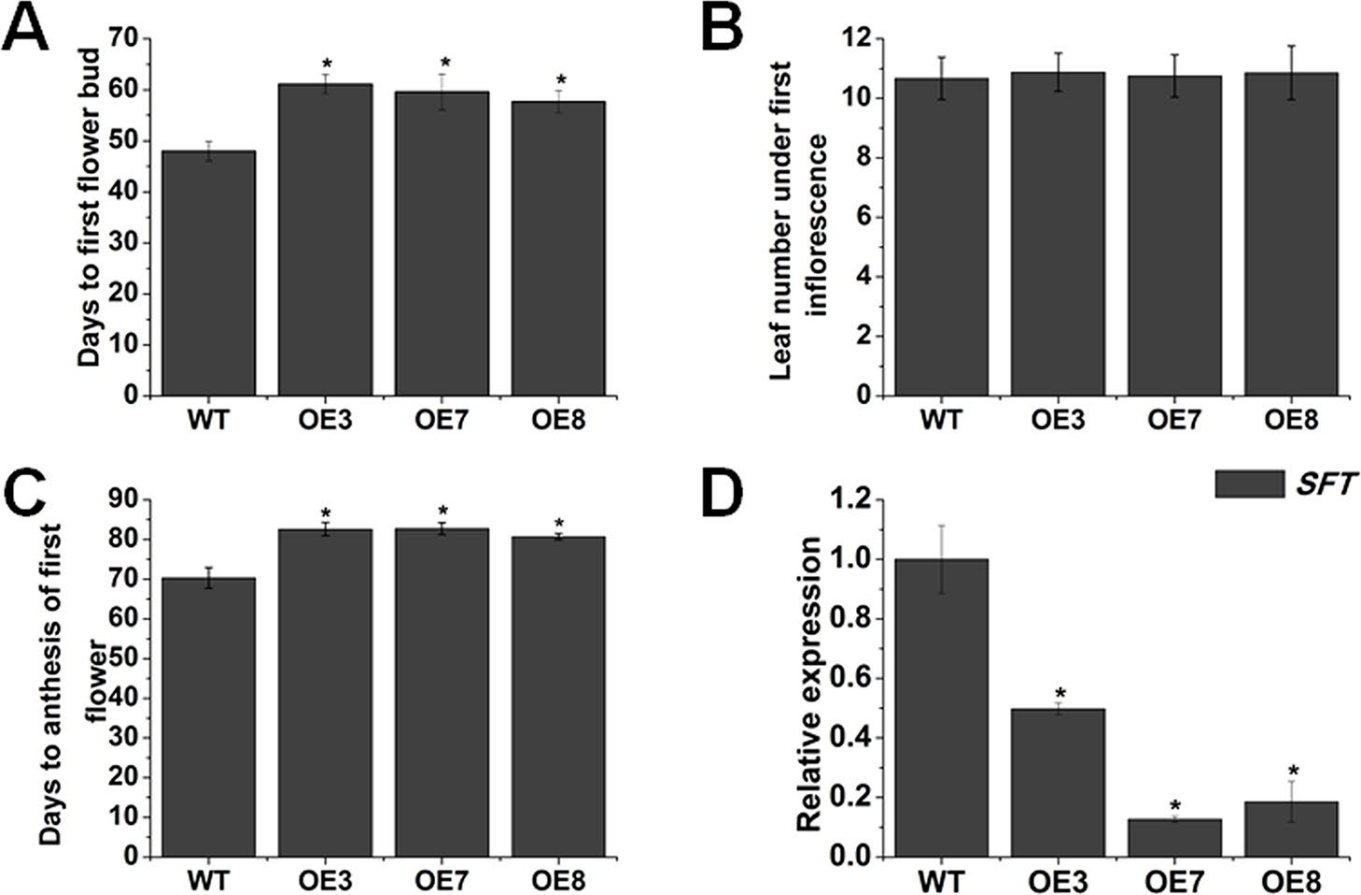
Overexpression of *SlOFP20* restrains the onset of ﬂowering. Analysis of the days to ﬁrst ﬂower bud **(A)**, leaf number under ﬁrst inﬂorescence **(B)** and **(C)** days to anthesis of ﬁrst ﬂower of WT and transgenic plants. Error bars show the standard error between three biological replicates with eight plants for each replicate performed. **(D)** Expression analysis of *SFT* in WT and transgenic plants. Each value represents the mean ± SE of three biological replicates. Asterisks indicate a signiﬁcant difference (*P* < 0.05).

Nearly all BR-deﬁcient and BR-insensitive mutants display late-ﬂowering phenotype (Li et al., 2010). Here, we also observed that overexpression of *SlOFP20* obviously restrained the initiation of ﬂower buds. To quantify this phenotype in more detail, the related parameters, which are usually used to assess the ﬂowering time, were measured. For days to ﬁrst ﬂower bud emergence, the *SlOFP20*-OE transgenic plants generated ﬂower buds much later than those of WT plants. Flower buds arose at least 9 days later in overexpression lines than WT tomato plants in our growth conditions (**Figure 4A**). In addition, we also measured days to anthesis of ﬁrst ﬂower (**Figure 4C**), which were consistent with the days to ﬁrst ﬂower bud. However, another parameter, leaf numbers below the ﬁrst inﬂorescence, showed no obvious discrepancy between WT and *SlOFP20*-OE transgenic plants (**Figure 4B**). *SFT is* a central regulator to control flowering time in tomato (Lifschitz and Eshed, 2006; Shalit et al., 2009). We supposed that overexpression of *SlOFP20* delayed flowering through the *SFT* pathway. Thus, the expression level of *SFT* was determined, which was significantly reduced in *SlOFP20*-OE plants when compared with WT tomato plants (**Figure 4D**).

Moreover, 10 µM 24-epi BR (eBR) was used to treat WT and *SlOFP20*-OE plants, and as a result, the leaf angle of WT plants was significantly increased, while that of the OE plants was only slightly increased. This implies that *SlOFP20*-OE plants displayed a decreased sensitivity to eBR treatment (**Supplementary Figure S1**). Furthermore, exogenous application of 50 µM GA3 could partially restore defects in plant height (**Supplementary Figure S2**). Combining the results of our current paper with previous results (Wang et al., 2007; Yang et al., 2018), we suppose that ectopic expression of *SlOFP20* may exhibit typical BRs-insensitive and GA-deﬁcient phenotypes.

### Overexpression of *SlOFP20* in Tomato Promotes Chlorophyll Accumulation in Leaves

**Figure 5:**
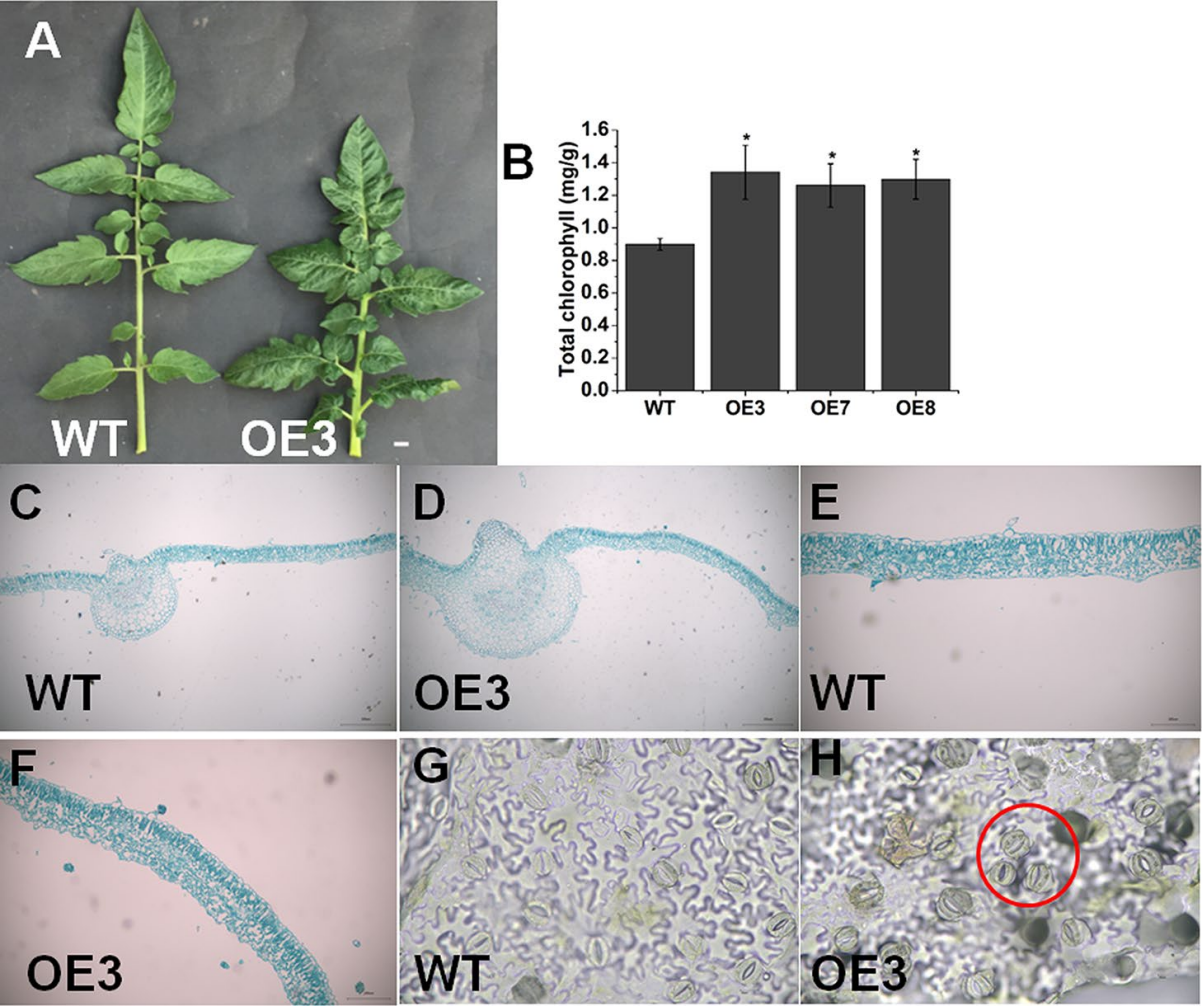
Overexpression of *SlOFP20* alters leaf growth. **(A)** Comparison of the leaves of WT and OE3 plants. Bar = 1 cm. **(B)** Total chlorophyll content in mature leaves of WT and transgenic plants. Each value represents the mean ± SE of three biological replicates. Asterisks indicate a signiﬁcant difference (*P* < 0.05). **(C**–**F)** The leaf structure of WT and OE3 mature leaves. Bar = 100 μm. Stoma morphology of WT **(G)** and OE3 **(H)** leaves. Bar = 100 μm.

We found that *SlOFP20*-OE plants exhibited shorter compound leaves and darker green when compared with WT plants (**Figure 5A**). The chlorophyll contents of leaves were measured, indicating that overexpression lines had more chlorophyll compared to WT plants (**Figure 5B**). Moreover, *SlOFP20*-OE transgenic lines showed thicker leaves, and the anatomical study confirmed the increased thickness of leaf blade in *SlOFP20*-OE lines relative to WT plants (**Figures 5C–F**). We also performed hand sectioning to observe stomata morphology and stomata clustering event was found in *SlOFP20*-OE transgenic lines (**Figures 5G, H**), which was closely related to BR deficiency and BR signaling deficiency (Kim et al., 2012).

**Figure 6:**
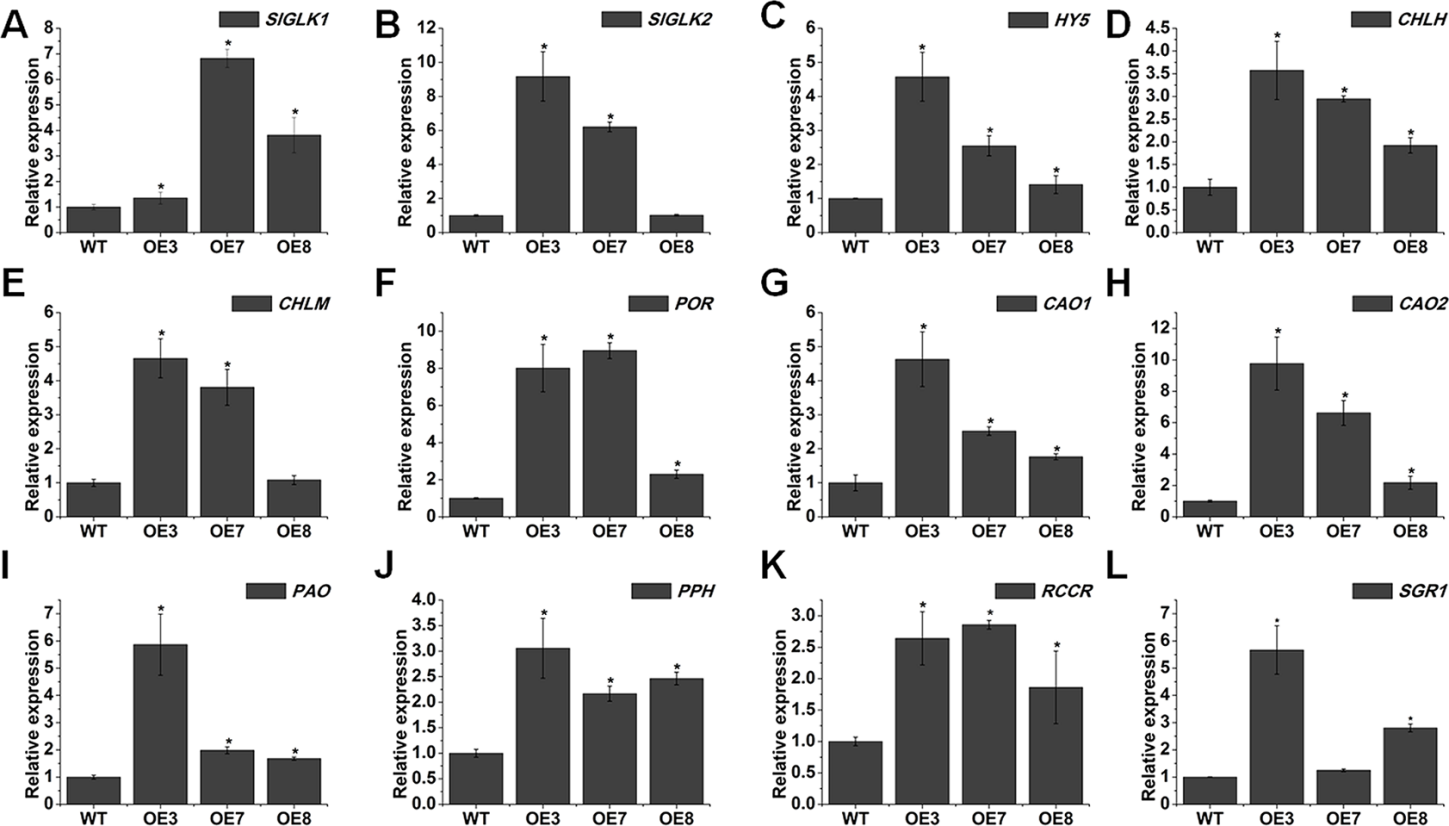
Overexpression of *SlOFP20* alters the expression of chloroplast development- and chlorophyll metabolism-related genes. **(A**–**C)** Quantitative RT-PCR analysis of the expression levels of *SlGLK1*, *SlGLK2*, and *HY5*. **(D**–**H)** Expression analysis of genes involved in the chlorophyll biosynthesis. in the mature leaves of WT and transgenic plants **(I**–**L)** qRT-PCR analysis of chlorophyll degradation-related genes in the mature leaves of WT and transgenic plants Each value represents the mean ± SE of three biological replicates. Asterisks indicate a signiﬁcant difference (*P* < 0.05).

To reveal the possible molecular mechanism of chlorophyll accumulation in *SlOFP20*-OE dark green leaves, we analyzed the expression levels of genes related to chlorophyll accumulation and chloroplast development in mature leaves of WT and *SlOFP20*-OE lines. GLK transcription factors are involved in regulating the chloroplast and chlorophyll levels (Waters et al., 2008). There are two *GLKs* existing in tomato, namely *SlGLK1* and *SlGLK2*. The expression of both was significantly increased in *SlOFP20*-OE plants (**Figures 6A, B**). We also found the upregulation of *LeHY5* in *SlOFP20*-OE transgenic plants (**Figure 6C**). Because *LeHY5*-RNAi plants show defects in light responses, including inhibited seedling photomorphogenesis, loss of thylakoid organization, and reduced carotenoid accumulation (Liu et al., 2004), the observed phenotype in *SlOFP20*-OE transgenic plants could be reasonable.

To clarify whether *SlOFP20* affected chlorophyll metabolism, transcript levels of chlorophyll biosynthesis genes were detected. Five structural genes in the chlorophyll synthesis pathway, magnesium chelatase H subunit (*CHLH*), Mg protoporphyrin IX methyltransferase (*CHLM*), protochlorophyllide reductase (*POR*), and chlorophyllide a oxygenase (*CAO1* and *CAO2*), were dramatically up-regulated in *SlOFP20* transgenic lines (**Figures 6D–H**).

To determine whether *SlOFP20* is involved in chlorophyll degradation, the expression levels of three chlorophyll degradation genes, pheophytin pheophorbide hydrolase (*PPH*), pheophorbide an oxygenase (*PAO*), and red chlcatabolite reductase (*RCCR*) were examined. All of them were significantly increased in *SlOFP20*-OE plants when compared with WT plants (**Figures 6I–K**). *SGR1* is a chlorophyll degradation-related gene, and silencing of *SGR1* reduces chlorophyll degradation in tomato leaves and fruits (Hu et al., 2011). The expression level of *SGR1* was remarkably enhanced in *SlOFP20*-OE lines (**Figure 6L**).

### Overexpression of SlOFP20 Affects Sugar Metabolism

**Figure 7:**
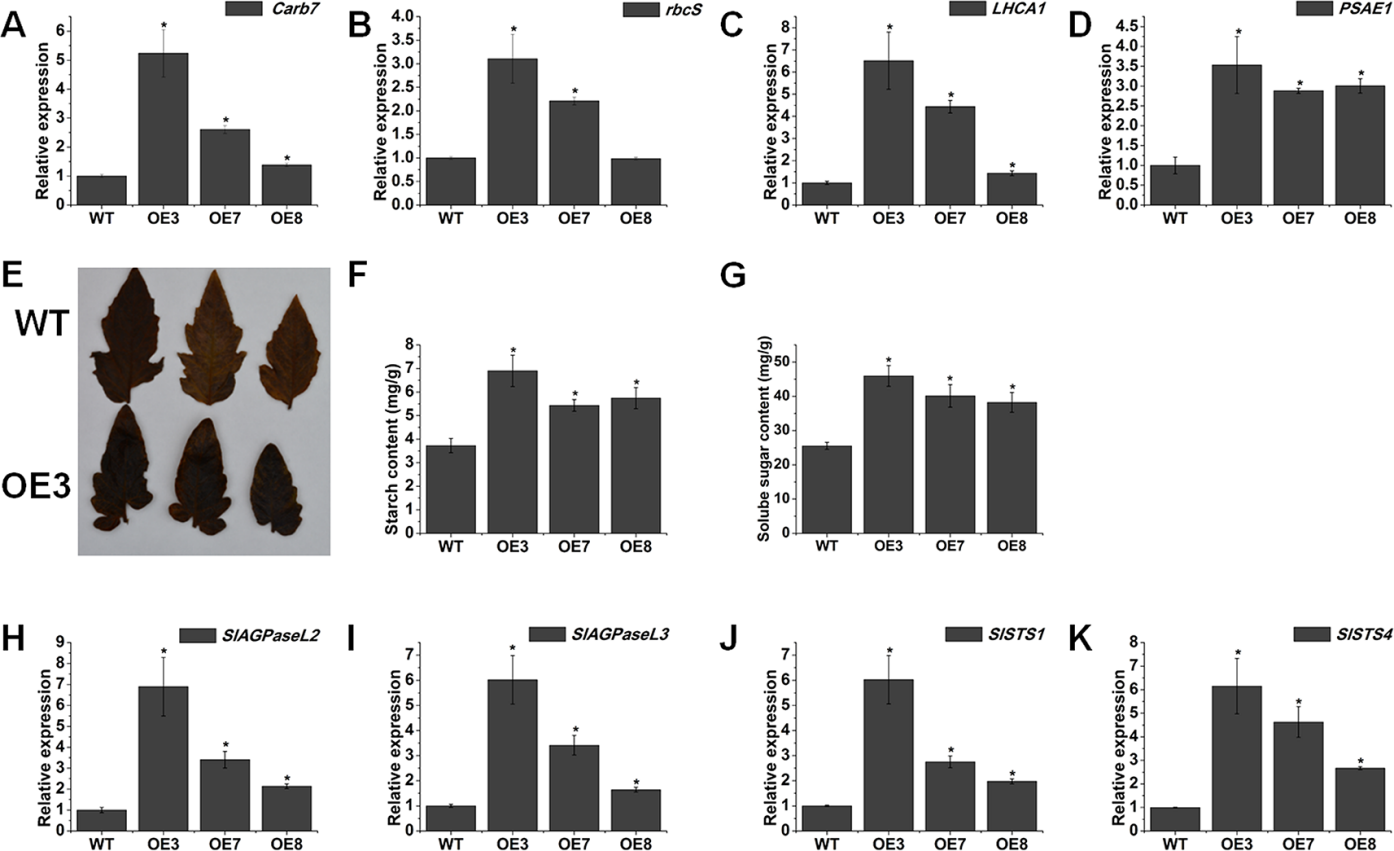
Ectopic expression of *SlOFP20* affects sugar metabolism. **(A**–**D)** The transcript levels of photosynthesis-related genes. **(E)** Starch I_2_/KI staining results for the WT and OE3 leaves. **(F**, **G)** Contents of starch and soluble sugar. **(H**–**K)** Expression analysis of genes involved in starch synthesis. Each value represents the mean ± SE of three biological replicates. Asterisks indicate a signiﬁcant difference (*P* < 0.05).

We wondered whether the increased chlorophyll content could confer higher photosynthetic performance in the *SlOFP20*-OE plants. To this end, we checked the expression level of photosynthesis-associated genes *cab7* and *rbcS* (John et al., 1995), and showed that their mRNAs were present at much higher levels in *SlOFP20*-OE mature leaves compared with WT mature leaves (**Figures 7A, B**). The mRNA accumulation of *LHCA1* and *PSAE1* which encode chlorophyll a/b-binding polypeptides and photosystem I reaction center subunit IV A respectively, was distinctively enhanced in *SlOFP20*-OE as compared to the WT (**Figures 7C, D**). We also detected the accumulation of sugars in the leaves of WT and *SlOFP20*-OE plants, which are the main products of chloroplast activity and photosynthesis. I_2_/KI staining was used to determine the accumulation of starch in the leaves of WT and *SlOFP20*-OE plants, indicating that *SlOFP20*-OE plants leaves accumulated much more starch than those of WT plants leaves (**Figure 7E**). Moreover, we measured the starch and soluble sugar content in the leaves of WT and *SlOFP20*-OE plants. These results suggested that *SlOFP20*-OE plants had significantly higher starch and soluble sugar contents when compared with WT plants (**Figures 7F, G**). To gain more insight into the mechanism of sugar metabolism in the *SlOFP20*-OE transgenic plants, we examined the expression patterns of starch biosynthesis genes. *SlAGPaseL2* and *AGPaseL3* encode the most important enzymes in starch synthesis that catalyse the first step of the reaction. *SlSTS1* and *SlSTS4* encode the starch synthases which catalyse the second step of starch synthesis. These results showed that all of these genes were distinctly induced in the mature leaves of *SlOFP20*-OE plants (**Figures 7H–K**).

### Transgenic Plants Overexpressing *SlOFP20* Display Prolonged Natural Senescence

**Figure 8:**
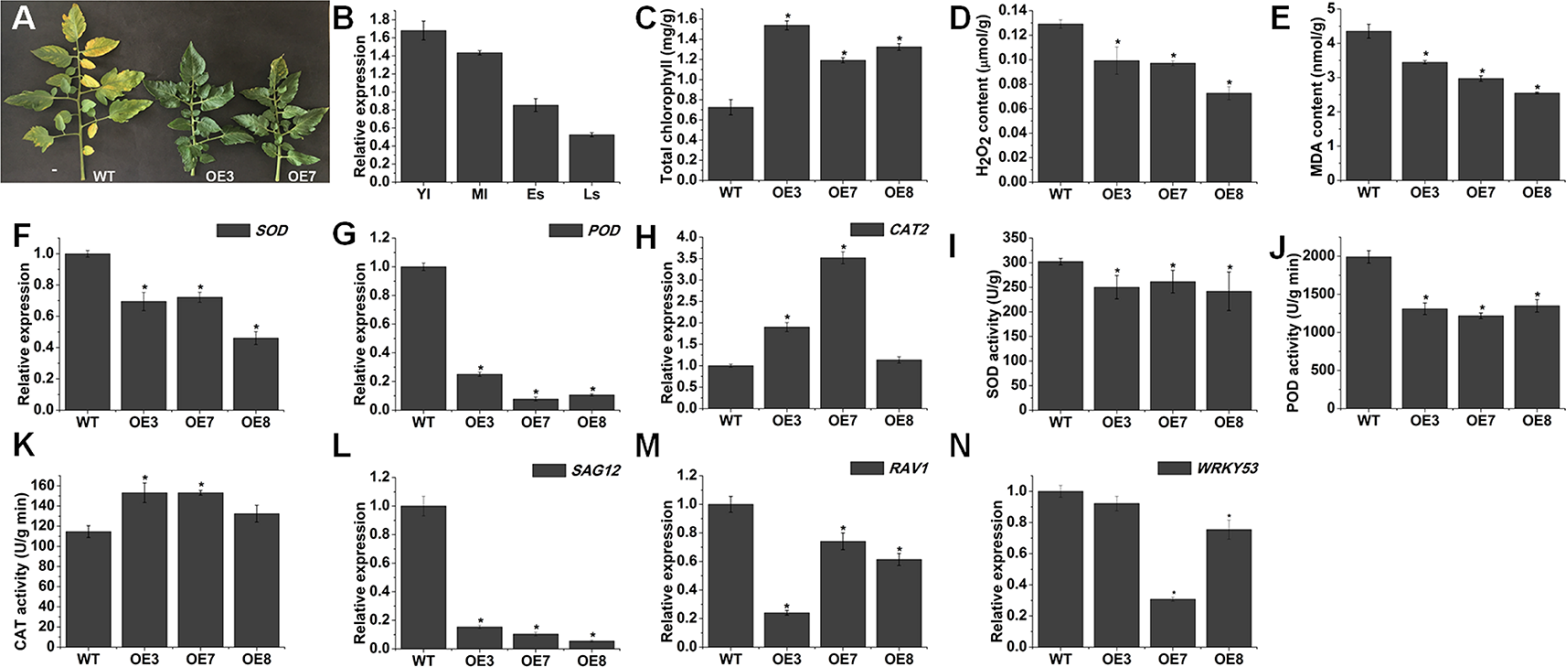
*SlOFP20* overexpression delays leaf senescence. **(A)** Phenotype of delayed leaf senescence in *SlOFP20*-OE lines. **(B)** The expression levels of *SlOFP20* in the leaves at different developmental stages: young leaves (YL), mature leaves (ML), senescent leaves (SL), and late senescent leaves (LS). **(C**, **D)** Comparison of H_2_O_2_ and MDA Contents in the senescent leaves of WT and *SlOFP20*-OE plants. **(F**–**H)** Comparison of transcript levels of *SOD*, *POD*, and *CAT2* in the senescent leaves of WT and transgenic plants. **(I**–**K)**. Comparison of SOD, POD, and CAT activities in the senescent leaves of WT and transgenic plants. **(L**–**N)** Expression of senescence-related genes (*SAG12*, *RAV1*, and *WRKY53*) in the senescent leaves of WT and transgenic plants. Each value represents the mean ± SE of three biological replicates. Asterisks indicate a signiﬁcant difference (*P* < 0.05).

**Figure 9:**
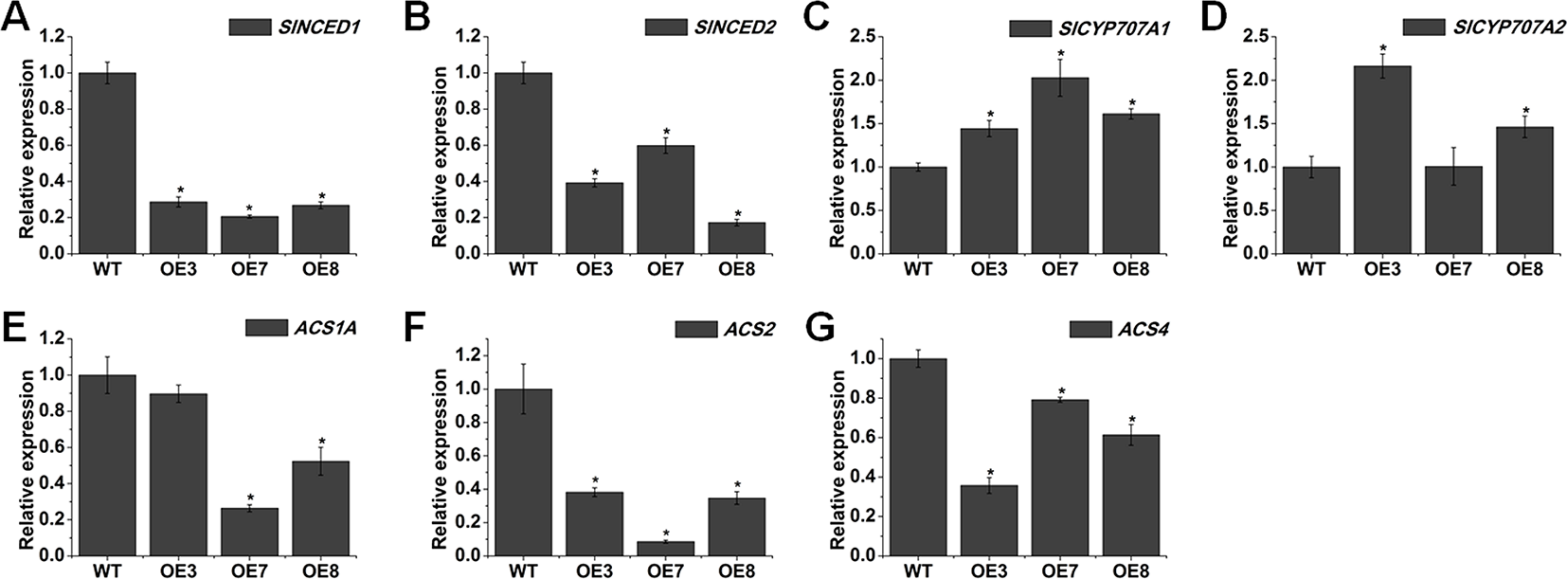
Overexpression of *SlOFP20* leads to the changes of abscisic acid (ABA)- and ethylene-related genes. **(A**, **B)** The expression of ABA biosynthesis genes (*SlNCED1* and *SlNCED2*) between WT and *SlOFP20*-OE lines. **(C**, **D)** The expression of ABA degradation genes (*SlCYP707A1* and *SlCYP707A2*) between WT and *SlOFP20*-OE lines. **(E**–**G)** The expression of ethylene biosynthesis genes (*ACS1A*, *ACS2*, and *ACS4*) between WT and *SlOFP20*-OE lines. Each value represents the mean ± SE of three biological replicates. Asterisks indicate a signiﬁcant difference (*P* < 0.05).

We found some delayed senescence in *SlOFP20*-OE transgenic lines when compared with WT plants during the whole life cycle (**Figure 8A**). To evaluate the contribution of *SlOFP20* in leaf senescence, we examined the expression level of *SlOFP20* at different developmental stages of WT tomato leaves, indicating that the mRNA accumulation of *SlOFP20* was gradually reduced since the beginning of senescence (**Figure 8B**). The visible appearance of leaf senescence is the degradation of chlorophyll, which is used as an index to assess leaf senescence. As shown in **Figure 8C**, there was a 1.64, 2.12, and 1.83 times higher total chlorophyll content in the leaves of OE3, OE7, and OE8 transgenic lines, respectively, when compared to WT leaves. To further explore this phenotype, we measure a series of physiological indicators related to leaf senescence. Enhanced production of reactive oxygen species (ROS) as well as MDA, a marker for lipid peroxidation, is characteristic of naturally senescent leaves (Dhindsa et al., 1981; Khanna-Chopra, 2012; Wang et al., 2013). The results suggested that the leaves of *SlOFP20*-OE lines had much less H_2_O_2_ and MDA contents than WT leaves (**Figures 8D, E**). Moreover, we also examined the mRNA accumulation of oxidative stress-related genes, which encode SOD, POD, and CAT, respectively. The expression levels of *SOD* and *POD* were significantly reduced in *SlOFP20*-OE lines when compared with WT (**Figures 8F, G**). However, the expression level of *CAT2* in *SlOFP20*-OE plants was higher than in WT plants (**Figure 8H**). In addition, the activities of SOD, POD, and CAT were measured, the results suggested that the activities of SOD and POD were signiﬁcantly lower in transgenic lines than in WT plants (**Figures 8I, J**). However, a signiﬁcantly higher CAT activity was measured in transgenic lines (**Figure 8K**). To determine whether overexpression of *SlOFP20* mediates the expressions of senescence-mediated genes, *SAG12* was evaluated as a marker gene of leaf senescence (Weaver et al., 1998). RAV1 acts as a positive regulator of leaf senescence in *Arabidopsis* (Woo et al., 2010). *WRKY53* acts in a complicated transcription factor signaling network regulating senescence speciﬁc gene expression (Miao et al., 2004). The results showed that the expression level of *SAG12*, *RAV1*, and *WRKY53* was down-regulated in *SlOFP20*-OE lines (**Figures 8L–N**). Leaf senescence is closely linked to differential phytohormones signaling, such as ABA and ethylene. To understand whether *SlOFP20* modulates leaf senescence *via* phytohormone pathways, we analyzed the expression level of ABA biosynthesis genes (*SlNCED1* and *SlNCED2*) and degradation genes (*SlCYP707A1* and *SlCYP707A2*). Gene expression analysis revealed that the transcript abundance of *SlNCED1* and *SlNCED2* in *SlOFP20*-OE lines was much less than WT plants (**Figures 9A, B**). In contrast, both *SlCYP707A1* and *SlCYP707A2* were up-regulated in *SlOFP20*-OE lines (**Figures 9C, D**). We further evaluated ethylene synthesis genes, including *ACS1A*, *ACS2* and *ACS4*, and the results suggested that expression of all these three genes was substantially repressed in *SlOFP20*-OE lines when compared to WT plants (**Figures 9E–G**).

## Discussion

OVATE domain-containing proteins belong to a plant-speciﬁc and poorly characterized family of plant regulatory proteins (Hackbusch et al., 2005). Recent studies reveal that OFPs participate in a series of plant growth and developmental processes through modulating hormone signaling. *AtOFP1* overexpression in *Arabidopsis* generates dwarf phenotype through weakening GA synthesis (Wang et al., 2007). The function of OsOFP8 in plant growth and development is at least partly through the BR signaling pathway in rice (Yang et al., 2016). Rice OFP 6 controls lateral root growth and initiation *via* mediating auxin (Ma et al., 2017). OsOFP19 negatively modulates the BR response to adjust plant growth and development (Yang et al., 2018). These findings strongly support the fact that OFPs are involved in mediating hormone signaling, BR and GA in particular. BR and GA are the two most important hormones that determine plant height by regulating cell elongation. Mutants deﬁcient in either BR or GA display a dwarf stature. In our study, we observed that overexpression of *SlOFP20* in WT tomato significantly reduced plant height (**Figure 2F**). The diminished plant size in *AtOFP1* and *OsOFP19* overexpression lines could partially be attributed to the deﬁciency in gibberellin biosynthesis and the reduced BR response, respectively. Exogenous application of eBR suppresses the expression of *SlOFP20* (unpublished data), as well as GA_3_ (**Figure 1A**). Therefore, we speculated that the multiple phenotypes caused by *SlOFP20* overexpression may be closely related to BR and GA signaling.

Here, we found that overexpression of *SlOFP20* led to reduced cotyledon size (**Figure 2E**) and hypocotyl length (**Figures 2A, B**), as well as plant height (**Figure 2F**). Moreover, decreased length of primary root and reduced number of lateral roots were observed, in agreement with the earlier observations as to some partial deﬁciency in key BR biosynthetic gene *DWARF* (Li et al., 2016). The deﬁciency of BR biosynthesis or signaling results in upright leaves. LEAF and TILLER ANGLE INCREASED CONTROLLER (*LIC*) acts as a negative regulator in BR response, and its gain-of-function mutant displays upright leaves (Zhang et al., 2012). BR level is connected with the bud outgrowth and *DWARF* overexpression obviously increased the number of lateral branches (Li et al., 2016). *SlOFP20*-OE plants showed obvious erect leaves (**Figures 3A, B**), the leaf angles of *SlOFP20*-OE lines were much smaller than WT plants (**Figure 3C**), and the expression level of *XET4* positively related to leaf inclination was significantly reduced in *SlOFP20*-OE lines (**Figure 3D**). Moreover, we also examined the lateral bud number in WT and *SlOFP20*-OE plants, the results suggested that the lateral bud number in *SlOFP20*-OE lines was clearly reduced (**Figure 3E**), and the transcript accumulation of *BRC1b* gene, which inhibits the outgrowth of lateral branches, was significantly enhanced in *SlOFP20*-OE lines (**Figure 3F**). Late-flowering phenotypes are found in BR-deficient or BR-insensitive mutants in *Arabidopsis* (Domagalska et al., 2007), and mutant plants that are deficient in GA also exhibit a late-flowering phenotype (Davière and Achard, 2013). Based on our observation, overexpression of *SlOFP20* delayed the ﬂowering process, further statistical analysis confirmed this phenotype (**Figures 4A–C**), and the expression level of *SFT*, which encodes a major player in flowering time control of the day-neutral plant tomato, was distinctly inhibited in *SlOFP20*-OE lines (**Figure 4D**). Furthermore, we also determined the expression levels of BR- and GA- related genes, and the results suggested that these genes were significantly affected in *SlOFP20*-OE lines (unpublished data). In addition, our yeast two-hybrid experiments confirmed that SlOFP20, KNOX1, and GRAS41 can form a complex to directly repress BR signaling, which was consistent with the previous finding that OsOFP19, OSH1, and DLT can form a complex that ﬁnely modulates the balance between plant growth and development and BR signaling (Yang et al., 2018). *SlOFP20*, *KNOX1*, and *GRAS41* are homologous genes of *OsOFP19*, *KNOX1*, and *DLT* from rice (unpublished data). *SlOFP20*-OE plants displayed greatly reduced sensitivity to eBR treatment (**Supplementary Figure S1**), and exogenous GA_3_ can partially restore defects in cell elongation in plants overexpressing *SlOFP20* (**Supplementary Figure S2**), which is consistent with previous observations (Wang et al., 2007; Yang et al., 2018). Based on this, we would like to conclude that ectopic expression of *SlOFP20* results in typical BRs-insensitive and GA-deﬁcient phenotypes. The phenotypes produced by *SlOFP20* overexpression resembled those of *AtOFP1*-OE and *OsOFP19*-OE plants, indicating that these homologous genes share similar functions in different plant species, most possibly as well as underlying molecular mechanisms. Besides, we found that overexpression of *SlOFP20* in tomato plants enhanced the accumulation of chlorophyll in leaves (**Figure 5A**), and retarded leaf senescence (**Figure 8A**), which have not been studied in other OFPs.

Our knowledge of the complex regulatory mechanisms of chloroplast development and chlorophyll metabolism is very limited. In this report, we speculated that *SlOFP20* may positively regulate chloroplast development and chlorophyll metabolism. *SlOFP20*-OE lines showed significantly higher levels of chlorophyll content when compared with WT plants. To investigate the mechanism of chlorophyll accumulation and chloroplast development in the mature leaves of *SlOFP20*-OE lines, we analyzed gene expression levels in the mature leaves of *SlOFP20*-OE lines and WT plants. *HY5* and *GLKs* promote the expression of many genes related to chloroplast development (Kobayashi et al., 2012; Powell et al., 2012; Pogson et al., 2015). The expression levels of *HY5*, *SlGLK1* and *SlGLK2* were distinctly enhanced by overexpressing *SlOFP20* in WT tomato (**Figures 6A–C**). Moreover, we also explored the effects of *SlOFP20* overexpression on chlorophyll metabolism. These five chlorophyll biosynthetic genes, *LHCH*, *LHCM*, *POR*, *CAO1*, and *CAO2* were sharply boosted in transgenic lines (**Figures 6D–H**), whereas the chlorophyll degradation genes, *PPH*, *PAO*, *RCCR*, and *SGR1* were also significantly increased (**Figures 6I–L**), implying that the accumulation of chlorophyll in *SlOFP20*-OE plants may activate the chlorophyll degradation pathways. A number of studies have suggested that chlorophyll content may be a positive correlation with photosynthetic capacity. For example, overexpression of *SlARF10* results in a dark-green phenotype and positively affected photosynthesis in both leaves and fruit (Yuan et al., 2018). The enhanced chlorophyll content in *SlARF4* down-regulated fruits correlates with a higher photochemical efﬁciency compared with WT fruits (Sagar et al., 2013). The basic helix-loop-helix transcription factor *PHYTOCHROME INTERACTING FACTOR3* (*PIF3*) negatively regulates light responses, repressing light-mediated cotyledon expansion, opening, and chlorophyll biosynthesis in the dark (Liu et al., 2013). We speculate that the increased chlorophyll content may upgrade the photosynthesis in *SlOFP20*-OE lines. Therefore, the expression levels of two photosynthetic-related genes, *cab7* and *rbcS*, were checked, the results suggested that both of them were increased in *SlOFP20*-OE lines (**Figures 7A, B**). *LHCA1* and *PSAE1* which encode chlorophyll a/b-binding polypeptides and photosystem I reaction center subunit IV A respectively, the mRNA accumulation of them was distinctly enhanced in *SlOFP20*-OE when compared to WT (**Figures 7C, D**). Moreover, we also measured sugar content in the mature leaves of *SlOFP20*-OE and WT plants, as the main products of photosynthesis. As expected, *SlOFP20*-OE plants accumulated more starch and soluble sugar content than WT plants (**Figures 7E–G**). We also examined the expression patterns of starch biosynthesis genes, including *SlAGPaseL2*, *AGPaseL3*, *SlSTS1*, and *SlSTS4*, indicating that overexpression of *SlOFP20* increased the transcripts of all four genes in mature leaves (**Figures 7H–K**). These results suggested that *SlOFP20* may play an important role in chlorophyll and sugar metabolism.

Leaf senescence represents the final stage of leaf development. Its time of onset is thus a major determinant of crop yield and quality. Therefore, plasticity in the timing of leaf senescence and the delicate balance between the onset and extent of leaf senescence are essential for ecological success and crop yield (Ma et al., 2018). In this study, transgenic plants with enhanced expression of *SlOFP20* exhibit a delay of leaf senescence during typical age-dependent senescence (**Figure 8A**). The expression *SlOFP20* of in leaves collected at different developmental stages declined along with the onset of leaf senescence (**Figure 8B**), implying that it may play a negative regulatory role in this process. Consistent with the delayed-senescence phenotype, *SlOFP20*-OE plants retained more total Chl levels than WT plants during senescence (**Figure 8C**). Leaf senescence induces the expression level of many SAGs, such as hormone signaling, transcriptional regulation, and chlorophyll catabolism (Buchanan-Wollaston et al., 2003; Lim et al., 2007; Li et al., 2014; Li et al., 2017). The senescence specificity of *SAG12*, encoding a cysteine protease, makes this gene as a molecular marker to study the senescence process (Noh and Amasino, 1999). *WRKY53* overexpression, RNAi and knock-out lines showed accelerated and delayed senescence phenotypes, respectively, and exhibited altered expression levels of the target genes (Miao et al., 2004). Constitutive and inducible overexpression of *RAV1* caused premature leaf senescence (Woo et al., 2010). *SAG12*, *WRKY53*, and *RAV1* were down-regulated in *SlOFP20*-OE lines when compared with WT plants, which was consistent with the phenotype of delayed senescence (**Figures 8L–N**). Reactive oxygen species are continuously produced in plants as products of aerobic metabolism (Maurino and Flügge, 2008). Excess ROS accumulation leads to oxidative damage to thylakoid membranes and other cellular components (Maurino and Flügge, 2008). Previous studies showed that early leaf senescence is usually associated with excessive ROS (Han et al., 2014; Ren et al., 2018; Wang et al., 2018). Compared with *SlOFP20*-OE plants, WT plants accumulated more H_2_O_2_ (**Figure 8D**). Furthermore, the content of MDA, a product of ROS-induced membrane lipid peroxidation, was also increased in WT plants when compared with *SlOFP20*-OE plants (**Figure 8E**). ROS can lead to the activation of antioxidative enzymes in plants, including SOD, APX, CAT, POD, and GST, to alleviate oxidative damage (Apel and Hirt, 2004). Plant senescence can lead to the synthesis of antioxidative enzymes to remove ROS (Miller et al., 2010). Rice *dwarf and early-senescence leaf1* (*del1*) exhibits early leaf senescence with the accumulation of ROS, and the activities of SOD and POD are increased in *del1* plants (Leng et al., 2017). The activities of SOD and POD in *SlOFP20*-OE plants were lower than those of WT plants (**Figures 8I, J**). The activity of CAT was increased in *SlOFP20*-OE plants (**Figure 8K**). Moreover, the changes in transcript levels of *SOD*, *POD*, and *CAT2* agreed with the changes in activity (**Figures 8F–H**). Furthermore, numerous studies confirmed that ABA and ethylene play vital roles in leaf senescence (Gao et al., 2016; Ren et al., 2018; Tang et al., 2018). Here, we measured the transcript accumulation of ABA biosynthesis genes (*SlNCED1* and *SlNCED2*) and degradation genes (*SlCYP707A1* and *SlCYP707A2*). *SlNCED1* and *SlNCED2* were down-regulated in *SlOFP20*-OE plants (**Figures 9A, B**), and the *SlCYP707A1* and *SlCYP707A2* were up-regulated (**Figures 9C, D**). We also determined the mRNA levels of ethylene synthesis genes, including *ACS1A*, *ACS2*, and *ACS4*, and their expression levels were decreased in *SlOFP20*-OE plants (**Figures 9E–G**). Therefore, we inferred that overexpression of *SlOFP20* delays leaf senescence in relation to ABA and ethylene synthesis.

In summary, our data suggest that *SlOFP20* overexpression exert manifold effects on tomato growth and development through coordinating BR and GA signaling. In addition, we found that overexpression of *SlOFP20* promotes the accumulation of chlorophyll and sugar, and restrains the beginning of leaf senescence, which has not been reported in OFPs. Knockdown of *BRASSINOSTEROID INSENSITIVE1* (*BRI1*), coding for a BR receptor, results in reduced BR signaling and a dwarf stature with upright and dark-green leaves (Kir et al., 2015). BRs could promote ethylene biosynthesis *via* regulation of ACC synthase 5 (ACS5) and ACC oxidase activities (Hansen et al., 2009). BRs could also induce ABA biosynthesis (Xia et al., 2014; Zhou et al., 2014). The expression of ethylene biosynthesis genes and ABA biosynthesis genes was inhibited in *SlOFP20*-OE plants. We infer that these two phenotypes may be closely linked with the reduced BR response in *SlOFP20*-OE lines. Therefore, it is meaningful to decipher how SlOFP20 harmonizes the crosstalk between different hormones to control plant growth and development in the future.

## Data Availability Statement

All datasets for this study are included in the article/ **Supplementary Material**.

## Author Contributions

GC, QX, and ZH designed and managed the research work and improved the manuscript. SZ, XC, FL, PF, and GH performed the experiments. SZ wrote the manuscript.

## Funding

This work was supported by the National Natural Science Foundation of China (no. 31872121), and the National Natural Science Foundation of Chongqing of China (cstc2018jcyjAX0458).

## Conflict of Interest

The authors declare that the research was conducted in the absence of any commercial or financial relationships that could be construed as a potential conflict of interest.
