# Supplementary material for: Overexpression of SlOFP20 in Tomato Affects Plant Growth, Chlorophyll Accumulation, and Leaf Senescence
Source: Front Plant Sci. 2019 Nov 29;10:1510. doi: 10.3389/fpls.2019.01510 (PMC6896838; doi:10.3389/fpls.2019.01510)
Supplement: Supplementary file 1 [file DataSheet_1.docx]

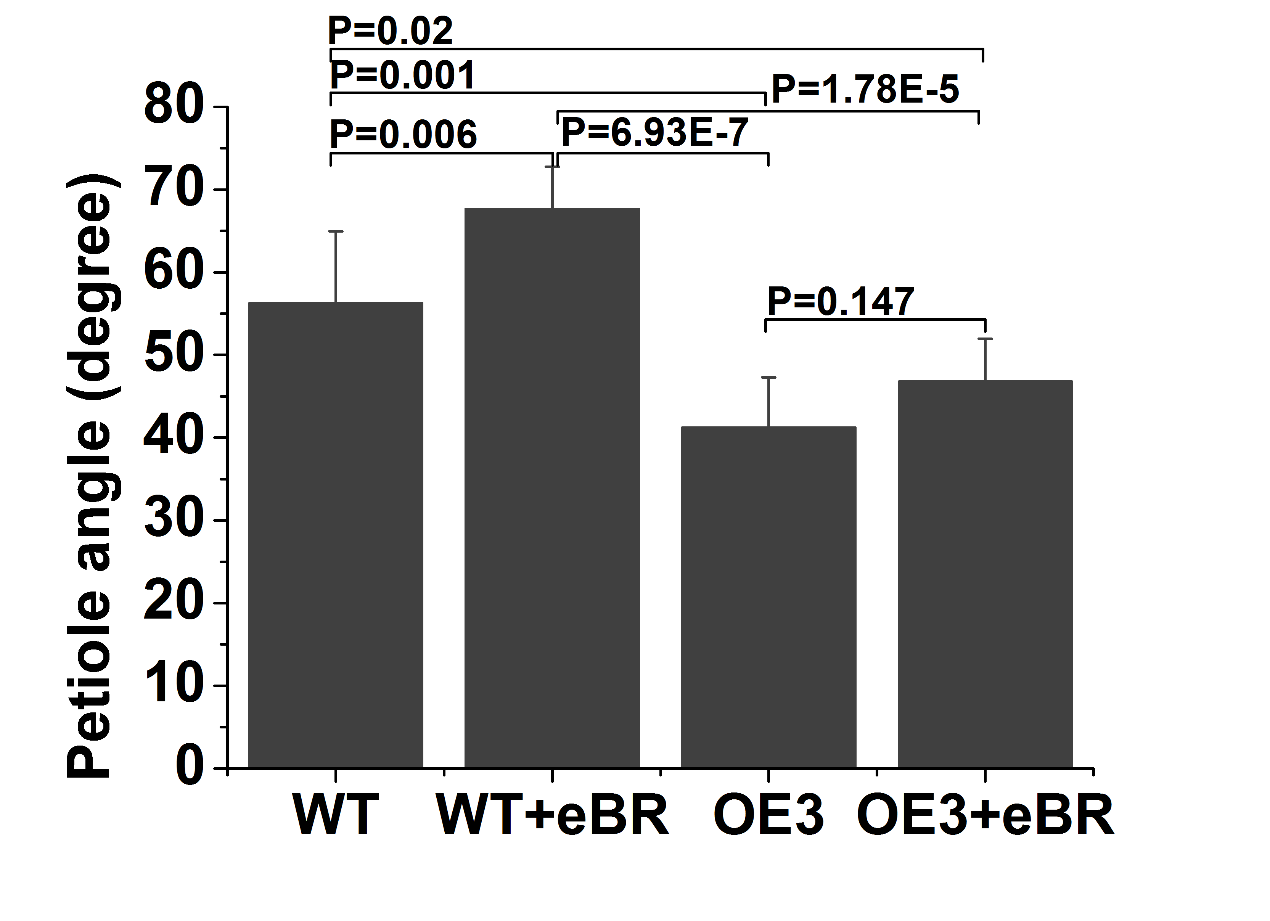


Fig. S1 Petiole angle as inﬂuenced by 10 μM eBR in WT and *SlOFP20*-OE plants. WT and *SlOFP20*-OE plants at the 6-leaf stage were sprayed with 10 μM eBR or water for two times with a 3-d interval. The third Petiole angle were measured. Data are the means ± SD of 8 plants for petiole angle. Data were analyzed by one-way analysis of variance (ANOVA), Fisher’s least significant difference t-test (LSD-t) was used for pairwise comparisons after the ANOVA. *P* < 0.05 was indicative of statistically significant difference.


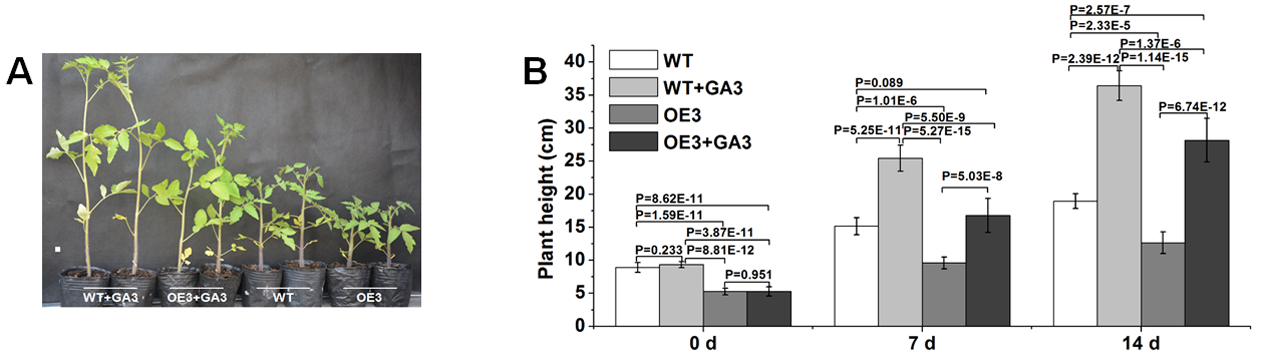


Fig. S2 The dwarf phenotype of *SlOFP20*-overexpressing plants was rescued by spraying with GA_3_.

(A) Pictures were taken 44 d after inoculation. The dwarf phenotype was rescued by spraying GA_3_ every 3 d starting from 30 d after inoculation. Bar = 1 cm (B) Plant height is displayed as the mean of 8 plants; error bars show SE (n=8). Data were analyzed by one-way analysis of variance (ANOVA), Fisher’s least significant difference t-test (LSD-t) was used for pairwise comparisons after the ANOVA. *P* < 0.05 was indicative of statistically significant difference.

**Table S1. All the primer sequences used in this study.**

| Primer name | Primer Sequence (5' →3') | Application |
| --- | --- | --- |
| *SlOFP20* | GCTCTAGA ACTAACTCCAATATCTCCCACTTTC | Overexpression  vector construton |
|  | CGAGCTC TCACGTAGAACATGAGTGTCTGA |  |
| *NPT II* | CTCAGAAGAACTCGTCAAGAAGG |  |
|  | GACTGGGCACAACAGACAATC | Positive transgenic plants detection |
| q*SlCAC* | CCTCCGTTGTGATGTAACTGG | Internal standard gene for quantitative RT-PCR |
|  | ATTGGTGGAAAGTAACATCATCG |  |
| q*SlOFP20* | AACGTCCACATCACTGAGCC | Gene expression detection |
|  | AGGGGTTGAATCAGGTTTCGTC |  |
| q*XET4* | TGGTGGTACTACGGCTGAAC |  |
|  | GACCGGTTCTATTCCCGAGG |  |
| q*BRC1b* | AGAAGGCCGAGGCGAAAA |  |
|  | GACCACGAGCGGTGTTGATC |  |
| q*SFT* | TTTTGGGCAAGAAATAGTGAGC |  |
|  | ATACACTGTTTGCCGACCTAAT |  |
| q*SlGLK1* | GAATTTTCCGTAAGCAGTGGTG |  |
|  | CTTCTCCTTGATTTAGGCTCGT |  |
| q*SlGLK2* | ACAATCGGAGGCGGAGGA |  |
|  | CAAGGAGTGCCTGGTACAAGAG |  |
| q*HY5* | TGCTAGTTTCGGGTGGATTG |  |
|  | CTAGGGAACGCTAGCAAAGG |  |
| q*SGR1* | ACTAGAAGGAAATGCAAGAAGAATCA |  |
|  | GCAACTTTCCTGGATGCTTTTC |  |
| q*CHLH* | GCTTTGGACCCACAGGCTAT |  |
|  | CTGTGCCAACGACTCTCCAT |  |
| q*CHLM* | AAGAAGGTGCCATTGTATCAG |  |
|  | CCATCCAAACTCTCCAAGTC |  |
| q*POR* | GCATCACATTTGCCTCCCTA |  |
|  | GAGTTCTTGTTCCAGCTCCAGTAC |  |
| q*CAO1* | GTGATCTTTCGTGGGGCTGA |  |
|  | CTCCCATCCGTGATAAGGGC |  |
| q*CAO2* | GGCATTTCGCTGAACAGGTTT |  |
|  | TGGCAATTGCTTTGCTCCAC |  |
| q*PAO* | CGAAATTGGCTTAGACGGCAT |  |
|  | ATCTGTCCATCATCTGGCGTT |  |
| q*PPH* | TGAGGTAACAGAACACCCTGC |  |
|  | TCATTCGACACCCAGTCAGTG |  |
| q*RCCR* | GTGGAGAATCGTCTTGGAGAGTC |  |
|  | CCGGTGGGTAAGTTGCAGTG |  |
| q*Cab7* | TAGACTTGCTATGTTAGCCGTTATG |  |
|  | TTCTGCTTCTCACTTGGGACTG |  |
| q*rbcS* | TGCTCAGCGAAATTGAGTACCTAT |  |
|  | AACTTCCACATGGTCCAGTATCTG |  |
| q*LHCA1* | GATGCCGGTCTACGTTGGAG |  |
|  | AATCCAAAATCTCCGGGGGC |  |
| q*PSAE1* | CAGAGACTCGTCGTAAGGGC |  |
|  | ACTCTCACCTTGGATCCCCT |  |
| q*PRE5* | CCTTCTCTCTTTGTCCATAACTTGTC |  |
|  | CCTGATGATTGTCTCGAACCG |  |
| q*SlAGPaseL2* | CCGAAGACCCCGTTCTACACT |  |
|  | TGCACCCATCATCATGGTATCC |  |
| *qSlAGPaseL3* | AAGGCGATGGCAGTAGACACAA |  |
|  | TGCAGTTGGAAAACGCCATC |  |
| q*SlSTS1* | GATTGGCTTCATCGGCAGAC |  |
|  | GTTCAATCTCCTGCTCAAACTCC |  |
| q*SlSTS4* | TGGAGCCTCAAAACGGGTTA |  |
|  | GCCGCATGACAGAAGAAACC |  |
| q*SOD* | GTCCACAGTCCATCATTGGAAGA |  |
|  | ATAGAAAACAAGGCTCAGCAGCT |  |
| q*POD* | CCACTCTCGTTGGAGGTCCAT |  |
|  | GGCAAAGCCAGAAATAAGCC |  |
| q*CAT2* | TTCTGCCCTTCTATTGTGGTTC |  |
|  | GTGATGAGCACACTTTGGAGC |  |
| q*SAG12* | CACACCCTAAGATTATGTCCTCCTC |  |
|  | CATCCACATTCCATTTGGTCCTTGA |  |
| q*RAV1* | ACAGAATAATACGAGGCGAACAA |  |
|  | TGCTACACTCAAATGCCAACAAC |  |
| q*WRKY53* | GGGGTGGCTCAACAGTACAATC |  |
|  | ATGTGGGCTGTGATTTTCTCTTC |  |
| q*SlNCED1* | CCCGATTTGGTATTCTGGATAAGTA |  |
|  | GAGACGGATTTCGGATAAAACACT |  |
| q*SlNCED2* | TGGTTTTCATGGGACATTCATTAGC |  |
|  | ATCTCCCTTCTCAACTCCCTATTCC |  |
| q*SlCYP707A1* | AGAAAGATGCCTATCACTAGCAGG |  |
|  | GTATTGGGTTTCGGCGCATT |  |
| q*SlCYP707A2* | GCGATTTGCTCGGGTCATT |  |
|  | TCTTGCTCTTCTGTGACAGCTTG |  |
| q*ACS1A* | AGAATAGTAATGAGTGGAGGAGCAA |  |
|  | ATTACAACTTTCACAAACAACTGGA |  |
| q*ACS2* | GAAAGAGTTGTTATGGCTGGTG |  |
|  | GCTGGGTAGTATGGTGAAGGT |  |
| q*ACS4* | GCTCGGAGGTAGGATGGTTTC |  |
|  | GTTCCTCTTCCATTGTGCTTGT |  |
